# Establishment and immune phenotyping of patient-derived glioblastoma models in humanized mice

**DOI:** 10.3389/fimmu.2023.1324618

**Published:** 2024-01-11

**Authors:** Longsha Liu, Thijs A. van Schaik, Kok-Siong Chen, Filippo Rossignoli, Paulo Borges, Vladimir Vrbanac, Hiroaki Wakimoto, Khalid Shah

**Affiliations:** ^1^ Center for Stem Cell and Translational Immunotherapy (CSTI), Harvard Medical School, Boston, MA, United States; ^2^ Department of Neurosurgery, Brigham and Women’s Hospital, Harvard Medical School, Boston, MA, United States; ^3^ Humanized Immune System Mouse Program, Ragon Institute, Massachusetts General Hospital, Harvard Medical School, Boston, MA, United States; ^4^ Department of Neurosurgery, Massachusetts General Hospital, Harvard Medical School, Boston, MA, United States; ^5^ Harvard Stem Cell Institute, Harvard University, Cambridge, MA, United States

**Keywords:** hGBM, GBM - multiforme, humanized mice, BLT humanized mice, flow cytometry, multiplex immunofluorescence staining

## Abstract

Glioblastoma (GBM) is the most aggressive and common type of malignant brain tumor diagnosed in adults. Preclinical immunocompetent mouse tumor models generated using mouse tumor cells play a pivotal role in testing the therapeutic efficacy of emerging immune-based therapies for GBMs. However, the clinical translatability of such studies is limited as mouse tumor lines do not fully recapitulate GBMs seen in inpatient settings. In this study, we generated three distinct, imageable human-GBM (hGBM) models in humanized mice using patient-derived GBM cells that cover phenotypic and genetic GBM heterogeneity in primary (invasive and nodular) and recurrent tumors. We developed a pipeline to first enrich the tumor-initiating stem-like cells and then successfully established robust patient-derived GBM tumor engraftment and growth in bone marrow-liver-thymus (BLT) humanized mice. Multiplex immunofluorescence of GBM tumor sections revealed distinct phenotypic features of the patient GBM tumors, with myeloid cells dominating the immune landscape. Utilizing flow cytometry and correlative immunofluorescence, we profiled the immune microenvironment within the established human GBM tumors in the BLT mouse models and showed tumor infiltration of variable human immune cells, creating a unique immune landscape compared with lymphoid organs. These findings contribute substantially to our understanding of GBM biology within the context of the human immune system in humanized mice and lay the groundwork for further translational studies aimed at advancing therapeutic strategies for GBM.

## Introduction

Glioblastoma (GBM) is the most prevalent primary malignancy of the central nervous system (CNS). Despite advances in understanding the development and progression of GBM, as well as subsequent treatments consisting of surgical resection (tumor debulking), chemotherapy, and radiation therapy, the prognosis for patients with GBM remains bleak (1). The urgent need for more effective therapies has led to the exploration of immunotherapy, a promising approach that harnesses the body’s immune system to fight cancer and achieved unprecedented success in malignancies such as melanoma (2–5). However, the development and testing of immunotherapies for GBM are hampered by the lack of reliable preclinical models (6), which are crucial for understanding the disease and predicting treatment efficacy.

Currently, preclinical research of immunotherapies for GBM relies on various methods, including *in vitro* assays, computational models, and animal models. *In vitro* assays, while useful for initial screening, oversimplify the tumor microenvironment and fail to capture the complex interplay between tumor cells, stromal cells, and the immune system (7). Despite their increasing sophistication, computational models are still unable to fully capture the complexity and dynamism of biological systems (8). Animal models have long been a cornerstone in preclinical research, providing valuable insights into disease pathophysiology and potential treatment efficacy. In the context of GBM, various types of animal models have been employed, including patient-derived xenografts (PDXs), genetically engineered mouse models (GEMMs), and syngeneic models (6, 9). While PDX models preserve the histological and genetic characteristics of human tumors, GEMMs and syngeneic models allow for the study of murine tumor-immune system interactions (7).

However, these models have several limitations. Mice, the most commonly used animals in preclinical research, have different immune systems (10) and tumor microenvironments (11, 12), which can lead to discrepancies in the efficacy and safety of immunotherapies between mice and humans. Most animal models in use are immunodeficient or immunocompromised to prevent the rejection of human cells or tissues, making it difficult to study immune responses (8). While the use of immune-competent mice offers promise, there still is a discrepancy between the human and murine immune systems. For example, the lymphocyte count in the blood circulation of a mouse is roughly twice as high as that in humans, while the neutrophil levels are significantly lower (13). Similar issues are encountered when using cell surface protein-targeting treatments, such as chimeric antigen receptor T cells or bivalent T cell engagers, where targets are inexistent in the murine setting or lack appropriate intracellular signaling (14). The intricate interplay between M1/M2 polarization in murine and human macrophages is another example that holds the potential to profoundly shape the tumor microenvironment where differences in macrophage polarization can exert diverse effects on tumor cells, including both pro-tumor activities such as angiogenesis and tissue remodeling, as well as potential suppression of these activities (15, 16). All these challenges have limited the predictive value of these mouse models, leading to a disconnection between preclinical findings and clinical outcomes.

The search for a more truthful representation of human immunopathology has led to the development of humanized mouse models created in immunodeficient mice engrafted with a functional human immune system. This allows for the investigation of human tumor-immune system interactions in mice (17). Among different humanized mouse models, the bone marrow-liver-thymus (BLT) model has the most functional immune system and allows the development of human T-cells that are able to recognize human HLA (18, 19). Furthermore, human B cells, macrophages, and dendric cells (DCs) are also functional, allowing an innate immune response to activate the adaptive immune system (20). Although the use of the BLT model with functional human immune systems is suggested to be a powerful and transformative methodology for translational cancer research moving forward, there is an urgent need to determine the feasibility of this modelling and the tumor-immune dynamics of the established tumor using patient-derived GBM lines.

Previously, we have isolated and characterized a large panel of glioma stem cell (GSC) lines, established from GBM-initiating cell-enriched cultures derived from human GBM specimens of newly diagnosed and recurrent tumors (21–24). Upon implantation of these GSC lines in mice, they retain patient-specific oncogenic molecular alterations, such as PTEN mutations and MGMT methylation, and the phenotypic hallmarks of GBM, such as extensive invasiveness or discrete nodular growth (21). In this study, we evaluated the feasibility of using the BLT mouse model to characterize patient-derived primary and recurrent GBM tumors. We first developed a pipeline to enrich the tumor-initiating stem-like cells and establish robust patient-derived GBM tumor engraftment and growth in BLT humanized mice. Furthermore, we explored the distinct immunophenotype that each patient-derived GBM line displays in the tumor microenvironment as well as in other lymphoid organs.

## Materials and methods

### Study design

The study was designed to evaluate whether hGBM lines grown in BLT humanized mice recapitulated human glioma development and progression, reflecting the genotypic and phenotypic changes seen in human GBMs. The objective was addressed by: (i) generating three distinct, imageable human-GBM lines that covered phenotypic and genetic GBM heterogeneity, (ii) analyzing immune profiles for human GBM tissue samples from which the GBM lines were generated, and (iii) utilizing flow cytometry and correlative immunofluorescence to assess immune-profiles in BLT models of the generated lines. All *in vivo* procedures were approved by the Institutional Animal Care & Use Committee at Brigham and Women’s Hospital (BWH) and Massachusetts General Hospital (MGH) (IACUC: 2019N000204). Patient brain tissue samples were obtained and analyzed under appropriate IRB approvals (IRB: 2005P001609) from Massachusetts General Hospital (MGH) and Dana Farber Cancer Institute (DFCI). The number of mice per group varied between experiments and is specified in the manuscript.

### Cell lines

The generation of patient-derived human GBM cell lines was previously described (21). Briefly, the resection specimens of newly diagnosed human GBM were obtained and used to create neurosphere cultures enriched for glioma stem cells (GSCs). GBM8, GBM18, and GBM31R were grown as spheres in Neurobasal medium (Life Technologies, Carlsbad, CA) supplemented with 3mM L-Glutamine (Life Technologies), B-27 supplement (Life Technologies), N-2 supplement (Life Technologies), 2 µg/ml heparin (Stem Cell Technologies, Vancouver, BC), 20 ng/ml EGF (Peprotech, Rocky Hill, NJ), and 20 ng/ml FGF (Peprotech), termed EF medium.

### Viral vectors and lentiviral transductions

For bioluminescence imaging, GBM cells were transduced with LV-Fluc-mCherry and selected by FACS sorting or Puromycin selection (1 µg/ml) in culture to generate GBM-FmC cells. mCherry expression was visualized by fluorescence microscopy.

### Western blot analysis

Cells were washed with PBS twice and then lysed with cold RIPA buffer (20 mM Tris-HCl pH 8.0, 137 mM NaCl, 10% glycerol, 1% NP-40, 0.1% SDS, 0.5% Na-deoxycholate, 2 EDTA pH 8.0) supplemented with protease and phosphatase inhibitors (Roche protease inhibitor cocktail; Phosphatase Inhibitor Cocktail I and Phosphatase Inhibitor Cocktail II from Sigma-Aldrich). Cells were then centrifuged at 4°C, 16,000 g, for 10 minutes. The protein concentrations were determined using a Bio-Rad protein assay kit. 6X SDS-sample buffer was added to the samples, which were then boiled for 3 minutes and resolved on SDS-polyacrylamide gel at a concentration of 30 µg per sample. SDS-polyacrylamide gels are transferred to the nitrocellulose membrane (Merck Millipore). The membranes were incubated with primary antibodies (**Supplementary Table S4**), followed by peroxidase-linked secondary antibodies developed with ECL (Thermo Fisher Scientific).

### Mouse passaging

To enrich and validate tumorigenic potential, hGBM-FmC tumor cells were initially implanted (1.5 × 10^5^ cells/mouse) into athymic nude mice (females, 6-8 weeks old with a provided 3-day facility acclimation period, 25-30g from Charles River Laboratories). Dissociated tumor cells suspended in 3 uL of 1X PBS were stereotactically implanted into the brains (right striatum, 2.5-mm lateral from Bregma and 2.5-mm deep) under anesthesia with ketamine-xylazine. Bioluminescence imaging was used to follow tumor growth and mice with established tumors were euthanized and brain tumor samples were grown in culture in EF medium supplemented with 0.5 × penicillin G/streptomycin sulfate/amphotericin B complex (Mediatech). The mouse passaged and dissociated cells were then implanted in both NOD-SCID and BLT mice, the former first used as a feasibility test before the same cells were implanted in BLT mice.

### Humanization in BLT mice

NOD/SCID/IL2Rγ^−/−^ (NSG) mice (The Jackson Laboratory) were housed in a pathogen-free facility at Massachusetts General Hospital. BLT humanized mice were generated as previously described (25). 8–12-week-old NSG mice were sub lethally (2-Gy) whole-body irradiated, anesthetized, and implanted with 1-mm^3^ fragments of human fetal thymus and liver tissue under the murine kidney capsule. Human fetal tissues (17 to 21 weeks of gestational age) were made available through Advanced Bioscience Resources (ABR; Alameda, CA). A total of 1 × 10^5^ autologous fetal liver tissue-derived human CD34^+^ hematopoietic stem cells (HSCs) were then injected intravenously within 6 h of tissue transplantation. Mice were maintained in microisolator cages and fed autoclaved food and water. Human immune reconstitution was then monitored over 12 to 20 weeks. Mice were generally considered reconstituted if greater than 50% of cells in the lymphocyte gate were human CD45^+^ and greater than 40% of these human CD45^+^ lymphocytes were human CD3^+^.

### Tumorigenicity studies in NOD-SCID and BLT mice

Dissociated GBMs that were mouse passaged once were stereotactically implanted into the brains (right striatum, 2.5-mm lateral from Bregma and 2.5-mm deep) of NOD-SCID mice (females, 6–8 weeks of age and 25–30 g from Charles River Laboratories) or BLT mice under anesthesia with ketamine-xylazine for tumorigenicity and characterization studies. Briefly, GBM8-FmC tumor cells (1.5 × 10^5^ cells/mouse), GBM18-FmC tumor cells (4 × 10^5^ cells/mouse) or GBM31R-FmC cells (5 × 10^5^ cells/mouse) were implanted, and bioluminescence imaging was used to follow tumor growth initially for NOD-SCID mice as an investigational pilot, followed by implantation into BLT mice. Mice were ultimately sacrificed when neurological symptoms became apparent, particularly as evidenced by significant motor function impairment, corresponding with a drastic expansion of the tumoral mass seen in most near-end-stage tumors. At which point, their brains are harvested for either tissue processing and immunofluorescence staining or flow cytometry (BLT only).

### Human tissue immunofluorescent multiplex staining and quantification

Paraffin-embedded human tissue sections were prepared, stained, imaged, and quantified by the CIO Tissue Biomarker lab, Dana Farber Cancer Institute. For multiplex staining, the following antibodies were used: CD11c-Opal570, CD3-Opal520, CD68-Opal780, CD8-Opal480, PD1-Opal620, SOX2-Opal690. Staining was conducted on a BOND RX fully automated stainer (Leica Biosystems). 5-μm thick sections of FFPE tissue were baked for 3 hours at 60°C before being loaded into the BOND RX. Slides were deparaffinized (BOND DeWax Solution, Leica Biosystems, Cat. AR9590) and rehydrated with a graded ethanol series. Antigen retrieval was performed in BOND Epitope Retrieval Solution 1 (pH 6) or 2 (pH 9), as shown below (ER1, ER2, Leica Biosystems, Cat. AR9961, AR9640) at 95°C. Slides were then consecutively stained with primary antibodies with an incubation time of 30 minutes per antibody. Afterward, anti-mouse plus anti-rabbit Opal Polymer Horseradish Peroxidase (Opal Polymer HRP Ms + Rb, Akoya Biosciences, Cat. ARH1001EA) was applied as a secondary label for 10 minutes. The signal for antibody complexes was labeled and visualized by their corresponding Opal Fluorophore Reagents (Akoya) by incubating the slides for 10 minutes. Opal Fluorophore 780 was paired with a TSA-DIG amplification. This pairing ensures an analyzable signal. Slides were incubated in Spectral DAPI solution (Akoya) for 10 minutes, air dried and mounted with Prolong Diamond Anti-fade mounting medium (Life Technologies, Cat. P36965). They were then stored in a light-proof box at 4°C prior to imaging. The target antigens, antibody clones, catalog numbers, dilutions for markers, diluents, and antigen retrieval details are listed in **Supplementary Table S1**.

Imaging was performed using the PhenoImager multispectral imaging platform (Akoya Biosciences, Marlborough, MA). Each slide was scanned at 20x resolution as whole-slide scan images. These images were then opened with Phenochart viewing software (Akoya Biosciences) where 4-6 20x regions of interest (ROIs) were selected. Once ROI selection was complete and approval from a pathologist (Scott J Rodig) was achieved, the images were spectrally unmixed and analyzed within Inform 2.6 (Akoya Biociences). Each analyzable ROI was segmented and quantified for expression of single and co-expressing markers utilizing the Inform analysis tools. Data tables were exported from Inform and run through a custom data extraction pipeline to obtain cell population densities (number of cells per mm2) for each marker and/or combinations of markers.

### Tissue processing and immunofluorescence staining

Tumor-bearing mice were perfused and brains were harvested, followed by coronal sectioning for histological analysis as previously described (26). Brain sections on slides were washed in PBS and then incubated for 2 hours in a blocking solution containing 11% v/v normal goat serum (#S-1000-20, Vector Laboratories), 0.9% v/v H_2_O_2_ and 0.2% v/v Triton X-100 in PBS. The sections were incubated overnight with primary antibodies (**Supplementary Table S2**) diluted in 2% v/v normal goat serum and 0.2% v/v Triton X-100 in PBS. The sections were washed in PBS for 3x20 minutes before incubating with the secondary. For standard fluorescence immunolabeling, goat anti-rabbit IgG conjugated to Alexa Fluor 488 (#A11008, Thermo Fisher Scientific) or 555 (#A21428, Thermo Fisher Scientific), goat anti-mouse IgG conjugated to Alexa Fluor 488 (#A21121, Thermo Fisher Scientific), or 555 (#A21127, Thermo Fisher Scientific) were diluted 1:500 in PBS containing 0.2% v/v Triton X-100 and incubated for 3 hours in a humidified light protected chamber. Alternatively, 1:500 diluted biotinylated goat anti-rabbit IgG (#BA-1000, Vector Laboratories), biotinylated goat anti-mouse IgG (#BA-9401, Vector Laboratories) or biotinylated goat anti-hamster IgG (#BA-9100, Vector Laboratories), incubated for one hour. The sections were then washed for 3x10 minutes with PBS before incubation with Streptavidin-Alexa Fluor 647 conjugate (#S21374, Thermo Fisher Scientific) was diluted 1:500 in 0.2% Triton X-100 in PBS for 1 hour. After secondary antibody and amplification incubation, the sections were washed for 3x10 minutes with PBS and incubated in 1:1000 dilution of 4’,6-diamidino-2-phenylindole (DAPI, #D1306, Thermo Fisher Scientific) in 0.2% Triton X-100 in PBS for 5 minutes. The sections were then washed for 3x10 minutes with PBS and coverslipped using ProLong Diamond antifade reagent (#P36961, Thermo Fisher Scientific) to be visualized with fluorescence microscopy. Fluorescence images were collected using a Zeiss Axio Observer Z1 LSM 710 BiG confocal microscope (Carl Zeiss) and captured with Zen 2012 software (Carl Zeiss). Images were pseudo-colored to permit overlay, cropping, sizing, and enhancement for contrast and brightness with Photoshop and Illustrator (Adobe Systems) or ImageJ (NIH). Automated cell counting of fluorescent images was performed with CellProfiler software (Broad Institute) (27).

### Flow cytometric analysis

After the BLI signal reached 5 x 10^5^ photons/second with 1 minute exposure time using the Perkin-Elmer IVIS Lumina system, the mice were sacrificed using ketamine-xylazine and diaphragm puncture. Subsequently, the mice were perfused with 10 ml of PBS by cardiac puncture, after which the brains were harvested, and the tumors were isolated. The tumor tissues were dissociated by mashing through a 100-μm strainer and washing with PBS. The samples were resuspended in PBS before staining with viable dye using Zombie UV Fixable Viability Kit (#423107, Biolegend). Cells were washed with FACS buffer (2% BSA and 5 mM EDTA in PBS) and blocked with both mouse and FcR blocker: FcR blocking reagent (Miltenyi Biotec) or Human TruStain FcX (#422301, BioLegend), respectively. Subsequently, the staining antibodies (**Supplementary Table S3**) were added in the PBMC-titrated concentrations and stained on ice for 1 hour. Following the staining, the cells were washed with FACS buffer, and analysis was performed using LSR Fortessa Cytometer (BD) with FACSDIVA and FlowJo v10 software.

### Statistical analysis

Data were expressed as mean ± SD and no statistical tests were required in this study.

## Results

### Patient-derived GBM tumor models are genetically and phenotypic distinct but have a similar immune compartment

**Figure 1:**
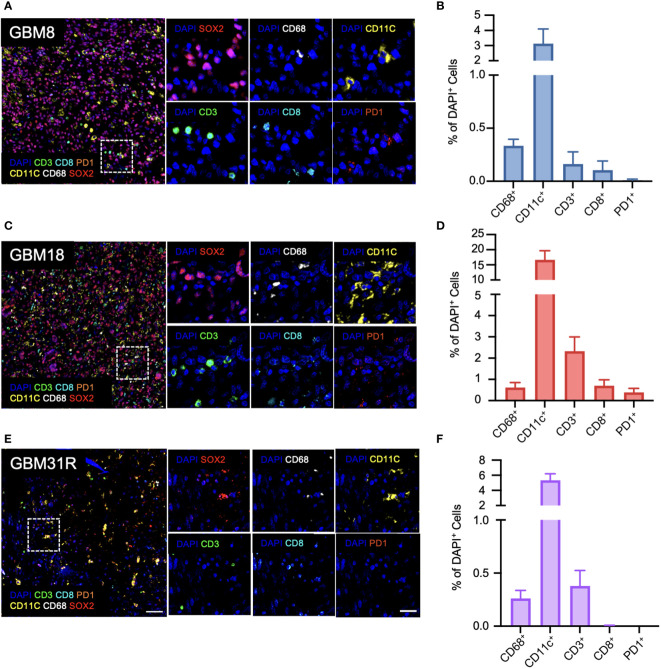
Immunological characterization of patient GBMs. **(A)** Representative immunofluorescent images of the stained markers (DAPI, CD11c, CD3, CD68, CD8, PD1, and SOX2) for GBM8 at a magnification of 20X. **(B)** Quantification of CD68-positive (Opal 780), CD11c-positive (Opal 570), CD3-positive (Opal 520), CD8-positive (Opal 480), and PD1-positive cells for GBM8 as a ratio of DAPI-positive cells. N=6. Mean ± SEM. **(C)** Representative immunofluorescent images of the stained markers (DAPI, CD11c, CD3, CD68, CD8, PD1, and SOX2) for GBM18 at a magnification of 20X. **(D)** Quantification of CD68-positive (Opal 780), CD11c-positive (Opal 570), CD3-positive (Opal 520), CD8-positive (Opal 480), and PD1-positive cells for GBM18 as a ratio of DAPI-positive cells. N=6. Mean ± SEM. **(E)** Representative immunofluorescent images of the stained markers (DAPI, CD11c, CD3, CD68, CD8, PD1, and SOX2) for GBM31R at a magnification of 20X. **(F)** Quantification of CD68-positive (Opal 780), CD11c-positive (Opal 570), CD3-positive (Opal 520), CD8-positive (Opal 480), and PD1-positive cells for GBM31R as a ratio of DAPI-positive cells. N=6. Mean ± SEM. Scale bar (zoom out) = 200µm; Scale bar (zoom in) = 50µm.

To establish a preclinical model facilitating the study of the interactions between human tumors and the immune system in mice, we initially focused on understanding the immune landscape of several patient GBMs. Three different GBMs, each possessing distinct genomic and phenotypic features (21, 23), were selected for this purpose: primary GBM8, primary GBM18, and recurrent GBM31R (**Table 1**). To characterize these GBMs immune-phenotypically, we employed multiplexed immunofluorescence (mIF), which allows simultaneous detection of multiple biomarkers. The markers used for this study were selected to provide a nuanced characterization of both myeloid and lymphoid components, enhancing insights into the intricate interplay within the tumor microenvironment and potential therapeutic or diagnostic targets. Specifically, the expression of SOX2 identifies glioma stem-like cells, contributing to our understanding of tumor-initiating and maintenance capabilities within the broader glioblastoma microenvironment. The expression of CD11c identified DCs whereas CD68 identified myeloid macrophages. Furthermore, within the lymphoid compartment, CD3 staining facilitated the detection of T lymphocytes, with CD8 immunofluorescence staining enabling a more precise evaluation of CD8-positive cytotoxic T cell presence. PD1 immunohistochemistry assessed exhausted T cell populations within the lymphoid compartment. For primary GBM8, the mIF analysis revealed that the tumor was largely comprised of SOX2+ glioma stem-like cells (74.5%, **Figure 1A**; **Supplementary Figure 1A**). Within the tumor microenvironment, myeloid cells were predominant, represented by CD11c+ DCs (3.13%, **Figures 1A, B**) and CD68+ macrophages (0.33%, **Figures 1A, B**), while T lymphocytes accounted for only 0.16% (**Figures 1A, B**). Comparatively, GBM18 exhibited a lower percentage of SOX2+ cells (50.6%, **Figure 1C**; **Supplementary Figure 1A**) with an immune microenvironment primarily composed of myeloid cells, including CD11c+ DCs (16.6%, **Figures 1C, D**) and CD68+ macrophages (0.62%, **Figures 1C, D**) with a smaller population of T lymphocytes (2.32%, **Figures 1C, D**). For recurrent GBM31R, a substantial proportion of the tissue appeared necrotic, perhaps reflecting treatment effects, with less than 5% of the cells being SOX2+ (**Figure 1E**; **Supplementary Figure 1A**). Nonetheless, myeloid cells remained dominant in the immune landscape where CD11c+ DCs (5.34%, **Figures 1E, F**) and CD68+ macrophages (0.26%, **Figures 1E, F**), and T lymphocytes constituted 0.38% of the cell population in GBM31R (**Figures 1E, F**). Across all three GBMs, PD1+ cells represented less than 0.5% of the cell population (**Figures 1A–F**). In summary, our findings indicate that although the percentages of different immune cell types vary among different GBMs, the overall number of immune cells within the tumors was relatively low. Moreover, the immune compartment in these GBMs was predominantly constituted by myeloid cells, with a smaller T lymphoid component. These insights are consistent with recent publications (28, 29) and represent a solid platform for the development of a preclinical model to investigate human GBM tumors in humanized mice.

**Table 1:** Pathological characterization of patient GBMs.

GBM #	*In vivo* phenotype		MGMT		IDH Mutation Status	Genomic Characterization
	Invasiveness	Pathological feature	Methylation	Protein		
8	Invasive	PNET-like component	M- Methylated	−	Wildtype	TERT promoter mutation, PIK3R1 mutation, MYCN amplification, PDGFRA amplification and MDM2 amplification
18	Nodular	Giant cell	M- Methylated	−	Wildtype	TERT promoter mutation, TP53 mutation, RB1 mutation
31R	Nodular	Conventional	U-Unmethylated	+	Wildtype	TERT promoter mutation; TP53 mutation; TSC2 mutation; RB1 homozygous loss

Table summarizing the pathological phenotype, methylation status, and genomic characterization of three patient GBMs (hGBM). All three models are IDH wildtype. M, Methylated; U, Unmethylated; +, positive; #, no.

### Enriched patient-derived GBM cells are able to form intracranial tumors in the BLT mice

**Figure 2:**
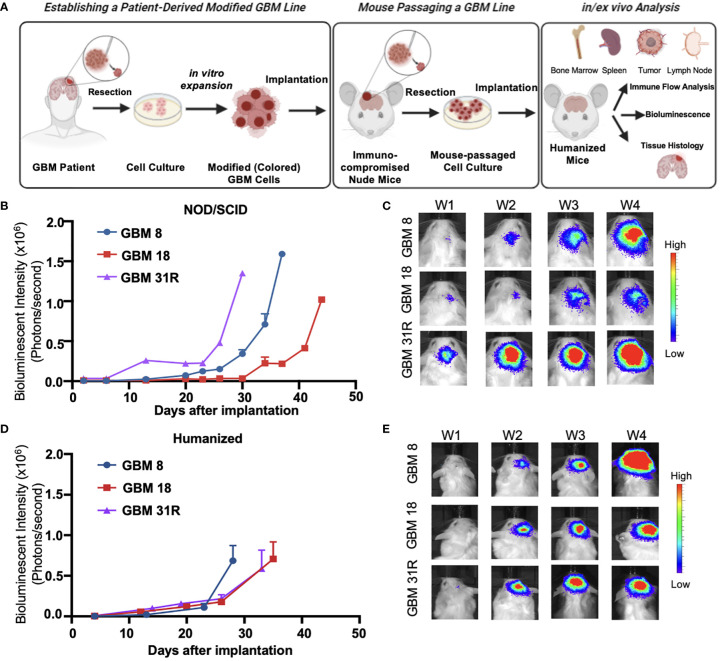
Characterization of growth of patient-derived GBM cell lines in different animal models. **(A)** Schematic overview of the experimental setup of establishing patient-derived GBM cell lines and the subsequent analysis in humanized mice. **(B)** Growth rate analysis of GBM cells expressing the luciferase reporter gene implanted in NOD-SCID mice, obtained by *in vivo* serial bioluminescence imaging. BLI flux was defined as photons/second. GBM8 (N = 2), GBM18 (N=2), GBM31R (N=1). Mean ± SEM. **(C)** Representative real-time bioluminescent IVIS images of GBM implanted NOD-SCID mice followed over time. **(D)** Growth rate analysis of GBM cells expressing the luciferase reporter gene implanted in BLT humanized mice, obtained by *in vivo* serial bioluminescence imaging. BLI flux was defined as photons/second. GBM8 (N = 5), GBM18 (N=5), GBM31R (N=5). Mean ± SEM. **(E)** Representative real-time bioluminescent IVIS images of GBM implanted BLT humanized mice followed over time.

Based on our previous work, we implemented a neurosphere culture protocol to enrich and expand GSCs derived from GBM8, GBM18, and GBM31R tumors (**Figure 2A**). These GSCs possess the ability to self-renew and generate orthotopic tumors that retain the primary tumor’s phenotype and genotype when implanted into immunodeficient mice (21, 22). To characterize the phenotypic and genotypic diversity among the cell lines, we performed a western blot analysis of the GBM cell lysates, revealing varying levels of p53, Akt, EGFR, p110a, Nectin1, and DR4/DR5 (**Supplementary Figure 1B**). We engineered these cells to express firefly luciferase (Fluc), for non-invasive, *in vivo* monitoring of tumor growth. To further enrich the GSCs *in vivo*, we implanted the GBM cells into the brains of athymic nude mice, subsequently harvested the tumors, expanded the cells through neurosphere culture, and evaluated tumor establishment in immunocompromised NOD/SCID mice and BLT mice. The growth rates of GBM tumors differed in NOD/SCID mice, with GBM31R exhibiting the fastest growth and GBM18 displaying the slowest growth (**Figures 2B, C**). Similarly, we observed tumor establishment in the brains of BLT mice using the same *in vivo* enriched cells although GBM8 exhibited the fastest growth while GBM18 and GBM31R displayed a similar growth rate (**Figures 2D, E**; **Supplementary Figure 1C**). These results demonstrate the feasibility of establishing humanized BLT mouse tumor models using patient-derived GBM cells, thus opening up an avenue for subsequent investigations of their immune profiles.

### Human immune cells infiltrate into the established GBMs in the BLT mice

**Figure 3:**
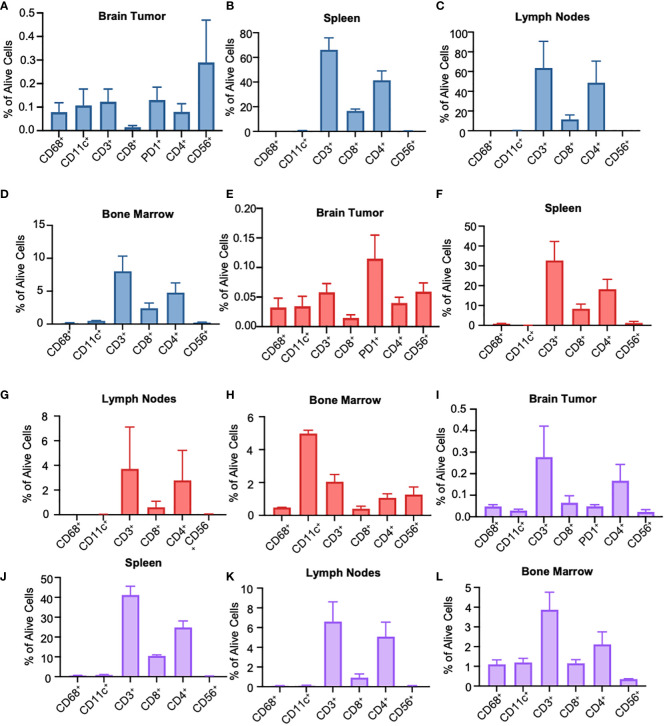
Flow cytometry characterization of the immune phenotype of human GBMs in different organ types of BLT humanized mice. **(A)** Flow cytometry analysis of tumor-infiltrating immunocytes as a fraction of living cells in BLT humanized mice bearing intracranial GBM8 tumors. N=3. Mean ± SEM. **(B)** Flow cytometry analysis of the spleen in humanized BLT mice bearing GBM8 tumors. Graphs represent single stained populations after life/death and hCD45 gating. N=3. Mean ± SEM. **(C, D)** Flow cytometry analysis of the deep cervical lymph nodes **(C)** and the bone marrow cells originating for the femur **(D)** in humanized BLT mice bearing GBM8 tumors. N=3. Mean ± SEM. **(E)** Flow cytometry analysis of tumor-infiltrating immunocytes as a fraction of living cells in BLT humanized mice bearing intracranial GBM18 tumors. N=3. Mean ± SEM. **(F)** Flow cytometry analysis of the spleen in humanized BLT mice intracranial GBM18 tumors. N=3. Mean ± SEM. **(G, H)** Flow cytometry analysis of the deep cervical lymph nodes **(G)** and bone marrow cells originating for the femur **(H)** in humanized BLT mice intracranial GBM18 tumors. N=3. Mean ± SEM. **(I)** Flow cytometry analysis of tumor-infiltrating immunocytes as a fraction of living cells in BLT humanized mice bearing GBM31R. N=3. Mean ± SEM. **(J)** Flow cytometry analysis of the spleen in humanized BLT mice implanted with GBM31R. N=3. Mean ± SEM. **(K, L)** Flow cytometry analysis of the deep cervical lymph nodes **(K)** and bone marrow cells originating for the femur **(L)** in humanized BLT mice implanted with GBM31R. N=3. Mean ± SEM.

**Figure 4:**
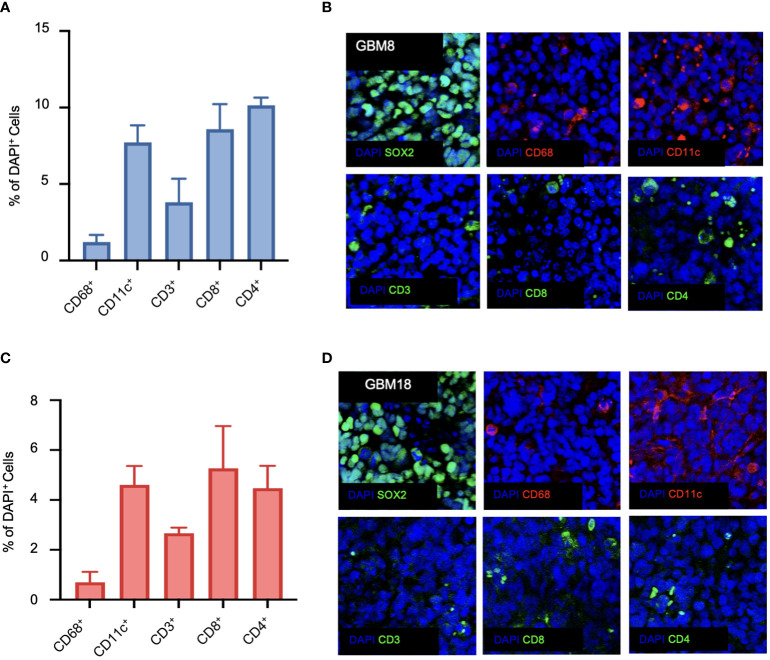
Immunofluorescence characterization of the immune phenotype of human GBMs in BLT humanized mice. **(A)** Quantification of CD68-positive, CD11c-positive, CD3-positive, CD8-positive, and CD4-positive cells for GBM8 as a ratio of DAPI-positive cells. N=6. Mean ± SEM. **(B)** Representative immunofluorescent images of the stained markers (DAPI, CD68, CD11c, CD3, CD8, and CD4) for GBM8 at a magnification of 20X. **(C)** Quantification of CD68-positive, CD11c-positive, CD3-positive, CD8-positive, and CD4-positive cells for GBM18 as a ratio of DAPI-positive cells. N=6. Mean ± SEM. **(D)** Representative immunofluorescent images of the stained markers (DAPI, CD68, CD11c, CD3, CD8, and CD4) for GBM18 at a magnification of 20X.

To understand the immune landscape of the GBM tumors in BLT mice, we harvested the tumor tissues from the brain, tumor-draining lymph nodes (TDLN), bone marrow (BM), and spleen from the mice, and performed flow cytometry using various human immune cell markers (**Supplementary Figure 2A**). For a better comparison across different staining techniques, we quantified the number of immune cells relative to the total number of living cells. In the BLT-derived GBM8 tumor, human immune cells were detectable, with CD56+ NK cells (0.29%), CD11c+ DCs (0.11%), CD68+ macrophages (0.08%), and T lymphocytes (0.12%) constituting the immune landscape (**Figure 3A**). Unlike the immune composition observed in tumor tissues, T lymphocytes were dominant in TDLN (63.6%), BM (8.03%), and spleen (66.2%), compared to CD11c+ DCs (TDLN: 0.27%; BM: 0.48%; spleen: 0.46%), CD68+ macrophages (TDLN: 0.01%; BM: 0.14%; spleen: 0.01%), and CD56+ NK cells (TDLN: 0.16%; BM: 0.25%; spleen: 0.39%) (**Figures 3B–D**). The BLT-derived GBM18 tumor exhibited an immune profile similar to GBM8, with a presence of CD56+ NK cells (0.06%, **Figure 3E**). For other tissues extracted from GBM18-bearing BLT mice, T lymphocytes were still the predominant immune population in TDLN (3.72%) and spleen (32.68%), but the CD11c+ DCs (4.98%) constituted predominantly in the BM (**Figures 3F–H**). Additional characterization of tumor markers for BLT-derived GBM8 and GBM18 was performed, including SOX2 (**Supplementary Figures 2B, C**), CD31 (**Supplementary Figures 2D, E**), and Ki67 (**Supplementary Figures 2F, G**). In contrast, the flow cytometric immune profile of the BLT-derived GBM31R tumor was dominated by T lymphocytes (~0.3%) rather than myeloid cells (**Figure 3I**). Yet, T lymphocytes were found to be dominant in other organs (TDLN: 6.61%; BM: 3.87%; spleen: 41.18%) from the mice bearing GBM31R (**Figures 3J–L**). Finally, we confirmed the presence of these human immune cells in the tumor through immunofluorescent staining of the tumor section in the GBM8 (**Figures 4A, B**) and GBM18 tumors (**Figures 4C, D**). These findings demonstrate the infiltration of human immune cells into the established intracranial GBMs and the immune compartment in other tissues of the BLT mice, setting the stage for future studies of human tumor-immune interactions in this preclinical model.

## Discussion

GBM poses a significant challenge due to its high prevalence in the central nervous system and the limited success of available treatments. Despite advances in understanding its development and the application of novel immunotherapies, the prognosis for GBM patients remains poor. A major hurdle in developing effective therapies is the lack of reliable models, creating a gap between laboratory findings and clinical outcomes. In this study, we addressed this issue by characterizing three patient-derived GBM tumors in humanized BLT mice, highlighting its relevance for a more human-oriented approach to studying GBM and its treatment.

The study commenced with an analysis of patient GBM tumors to uncover their genetic and phenotypic differences. The multiplexed immunofluorescence revealed the tumors’ unique phenotypic features. Among the analyzed tumors, GBM8 exhibited the highest proportion of SOX2+ glioma stem-like cells, followed by GBM18 and GBM31R. The immune microenvironment across all patient samples was characterized by dominant myeloid cells (CD11c+ DCs and CD68+ macrophages), with T lymphocytes constituting a smaller portion of the immune cell population. These findings are representative of GBM patients’ immune landscape, where myeloid cells represent the vast majority of non-cancerous cells in the tumor microenvironment, and in particular macrophages can account for up to 30% of the tumor mass (30). By contrast, tumor-infiltrating lymphocytes have been found to represent only about 2.5% of all cells in GBM, and usually consist of T-cells and to a lesser extent NK cells and B lymphocytes (31).

The isolated cells were enriched and expanded through neurosphere culture, and their capacity to establish tumors was assessed in immunocompromised mice. The grafted tumors displayed varied growth rates, highlighting the functional disparity among the GBM lines. This successful establishment of tumors in immunocompromised mice set the stage for subsequent experiments in humanized mouse models intended to examine the immune landscape within the established tumors.

Within these humanized models, human immune cells, encompassing CD56+ NK cells, CD11c+ DCs, CD68+ macrophages, and T lymphocytes, infiltrated the tumors. This infiltration was confirmed through immunofluorescent staining. Moreover, diverse lymphoid organs, including tumor-draining lymph nodes, bone marrow, and spleen, exhibited evidence of reconstitution by human immune cells. Although the types of immune cells detected were consistent between the tumor samples, we observed some differences in the distribution of the subpopulations between immunofluorescence and flow cytometry analysis. This can be due to the technical differences between the two assays. In particular, while flow cytometry processes the sample as a whole, irrespective of any spatial localization, immunofluorescence analyzes a specific region of the sample, represented by the slices. The spatial heterogeneity of the tumor could be reflected in these readout differences. Moreover, using different antibodies, buffers, and reference samples could have also contributed to the observed differences.

It is important to note, however, that there remain limitations to the BLT mouse model, including within the context of cancer research, that necessitate further development and exploration. For instance, these mice tend to develop Graft-versus-host disease (GvHD), a systemic inflammatory condition characterized by the transplanted graft’s immune cells attacking the host cells, restricting the experimental window to six months post-engraftment. However, BLT-humanized mice constructed with a C57BL/6 immunodeficient background, show resistance to GvHD (32). Another drawback lies in the need for advanced expertise, resources and experience for their production, along with the limited availability of required fetal tissues. Recently, a novel BLT-like humanized mouse model has emerged, utilizing non-autologous human cord blood-derived hematopoietic stem cells and human neonatal/pediatric thymus, partially dealing with these challenges. Furthermore, ongoing efforts are focused on improving BLT mice so that they develop a more complete human immune system as considerable challenges remain, such as cross-reactivity between rodents and humans, restricted development, differentiation, and migration of human hematopoietic stem cells, and the instability in the reconstitution of T-cells. The current widely used immunodeficient mouse model featuring an IL-2 receptor γ chain deletion results in incomplete development of mouse lymphoid organs, hampering the development of a robust humoral immune response. Constructing BLT-humanized mice through immunodeficient mouse models with necessary human transgenic factors and cytokines, or integrating the required human secondary lymphoid tissue, such as the spleen, could optimize human B cell development and overcome humoral immune response limitations in the model. Moreover, it necessitates mentioning that the BLT tumor microenvironment did not recapitulate the myeloid dominant TME seen in patients, highlighting a fundamental limitation in myeloid engraftment. To address this, NSG-SGM3 BLT mice, a model characterized by their transgenic expression of human stem cell factor (SCF), granulocyte macrophage colony-stimulating factor (GM-CSF), and interleukin-3 (IL-3) following engraftment of what normally characterizes BLT models (human hematopoietic stem cells, autologous fetal live, and thymic tissues), has demonstrated improved human B-cell development and represent a potential future avenue of investigation (33). Nevertheless, our findings provide compelling evidence that human immune cells can effectively infiltrate established GBM tumors within the BLT mouse model, thereby offering a more relevant representation of the human immune response *in vivo*, which was not attainable with traditional mouse models. These GBM models notably offer a platform to scrutinize how different components of the human immune system engage with the microenvironment of human tumors. Furthermore, our model provides a translational platform for testing novel therapeutics, including checkpoint blockade immunotherapy alone (34) or in combination with cytotoxic agents (35), allowing us to elucidate the variable effects observed in mouse immunocompetent models and human clinical trials within the complex immune landscape of GBM.

In conclusion, this study’s comprehensive exploration of patient GBM tumors and their interaction with the human immune system within the humanized BLT mouse model presents a promising avenue for advancing GBM research and immunotherapy development. This study confirms that patient-derived GBMs orthotopically established in humanized mouse models allow the recruitment and infiltration of variable human immune cells within the tumor, creating a unique immune landscape compared with lymphoid organs. Extending the study could involve directly implanting patient-derived samples into BLT mice to expedite the process, and faithfully represent the original tumor. These findings offer valuable insights into the intricate dynamics of GBM-immune interactions, opening new avenues for refining treatment strategies for this challenging malignancy and underscoring the necessity for a more faithful representation of the human immune system in preclinical models to enhance the precision and relevance of experimental outcomes in the pursuit of effective translational applications.

## Data availability statement

The original contributions presented in the study are included in the article/**Supplementary Material**. Further inquiries can be directed to the corresponding author.

## Ethics statement

Patient brain tissue samples were obtained and analyzed under appropriate IRB approvals from Massachusetts General Hospital (MGH) and Dana Farber Cancer Institute (DFCI). The studies were conducted in accordance with the local legislation and institutional requirements. The human samples used in this study were acquired from gifted from another research group. Written informed consent for participation was not required from the participants or the participants’ legal guardians/next of kin in accordance with the national legislation and institutional requirements. All *in vivo* procedures were approved by the Institutional Animal Care & Use Committee at Brigham and Women’s Hospital (BWH) and Massachusetts General Hospital (MGH). The study was conducted in accordance with the local legislation and institutional requirements.

## Author contributions

LL: Data curation, Formal analysis, Investigation, Methodology, Project administration, Validation, Visualization, Writing – original draft, Writing – review & editing. TS: Data curation, Formal analysis, Investigation, Methodology, Validation, Visualization, Writing – original draft. K-SC: Formal analysis, Investigation, Methodology, Resources, Validation, Visualization, Writing – review & editing, Data curation, Writing – original draft. FR: Formal analysis, Methodology, Validation, Visualization, Writing – original draft. PB: Methodology, Writing – original draft, Data curation, Investigation. VV: Methodology, Writing – original draft, Resources. HW: Methodology, Writing – original draft, Investigation, Supervision, Validation, Visualization, Writing – review & editing. KS: Conceptualization, Investigation, Methodology, Supervision, Validation, Visualization, Writing – review & editing, Formal analysis, Funding acquisition, Project administration, Resources.

## Supplementary material

Supplementary Figure 1 Characterization of the patient-derived GBM cell lines. **(A)** Quantification of SOX2-positive cells as a ratio of DAPI-positive cells for all samples. N=6. Mean ± SEM. **(B)** Western blot analysis showing the expression patterns of p53, p-p53, pAKT, AKT, EGFR, pEGFR, p110a, Nectin1, DR4, DR5, and IL-13Ra2 in the cell lysates prepared from hGBM lines after *in vitro* culturing. B-Actin was used as a loading control. **(C)** Images obtained from IVIS bioluminescence imaging were used to determine tumor growth of the BLT humanized mice for three patient-derived GBM cell lines (n=5 per group).

Supplementary Figure 2 Characterization of patient-derived GBM tumors in humanized BLT mice. **(A)** Representative flow cytometry plots showing the gating strategy of the immune phenotype of humanized BLT mice flow cytometry analysis. **(B)** Representative images of SOX2 single immunofluorescence staining in sections from GBM8 and GBM18 tumors implanted in humanized BLT mice at a magnification of 20X. **(C)** Quantification of SOX2-positive cells obtained from single immunofluorescence staining in sections from GBM8 and GBM18 tumors implanted in humanized BLT mice as a ratio of DAPI-positive cells. N=5. Mean ± SEM. **(D)** Representative images of CD31 single immunofluorescence staining in sections from GBM8 and GBM18 tumors implanted in humanized BLT mice at a magnification of 20X. **(E)** Quantification of CD31-positive cells obtained from single immunofluorescence staining in sections from GBM8 and GBM18 tumors implanted in humanized BLT mice as a ratio of DAPI-positive cells. N=5. Mean ± SEM. **(F)** Representative images of Ki67 single immunofluorescence staining in sections from GBM8 and GBM18 tumors implanted in humanized BLT mice at a magnification of 20X. **(G)** Quantification of Ki67-positive cells obtained from single immunofluorescence staining in sections from GBM8 and GBM18 tumors implanted in humanized BLT mice as a ratio of DAPI-positive cells. N=5. Mean ± SEM.
