# Corrigendum: Establishment and immune phenotyping of patient-derived glioblastoma models in humanized mice

**DOI:** 10.3389/fimmu.2024.1466387

**Published:** 2024-08-05

**Authors:** Longsha Liu, Thijs A. van Schaik, Kok-Siong Chen, Filippo Rossignoli, Paulo Borges, Vladimir Vrbanac, Hiroaki Wakimoto, Khalid Shah

**Affiliations:** ^1^ Center for Stem Cell and Translational Immunotherapy (CSTI), Harvard Medical School, Boston, MA, United States; ^2^ Department of Neurosurgery, Brigham and Women’s Hospital, Harvard Medical School, Boston, MA, United States; ^3^ Humanized Immune System Mouse Program, Ragon Institute, Massachusetts General Hospital, Harvard Medical School, Boston, MA, United States; ^4^ Department of Neurosurgery, Massachusetts General Hospital, Harvard Medical School, Boston, MA, United States; ^5^ Harvard Stem Cell Institute, Harvard University, Cambridge, MA, United States

**Keywords:** hGBM, GBM - multiforme, humanized mice, BLT humanized mice, flow cytometry, multiplex immunofluorescence staining

In the published article, there was an error in the Supplementary Figure 1 as published. While compiling the images for one of the panels, the same mouse image was erroneously used twice in the GBM18 group. The corrected images are now presented in the corrected Supplementary Figure 1.

The authors apologize for this error and state that this does not change the scientific conclusions of the article in any way. The original article has been updated.

