# Supplementary figures and images for: Establishment and immune phenotyping of patient-derived glioblastoma models in humanized mice
Source: Front Immunol. 2024 Jan 11;14:1324618. doi: 10.3389/fimmu.2023.1324618 (PMC10808686; doi:10.3389/fimmu.2023.1324618)

Supp. Figure 1

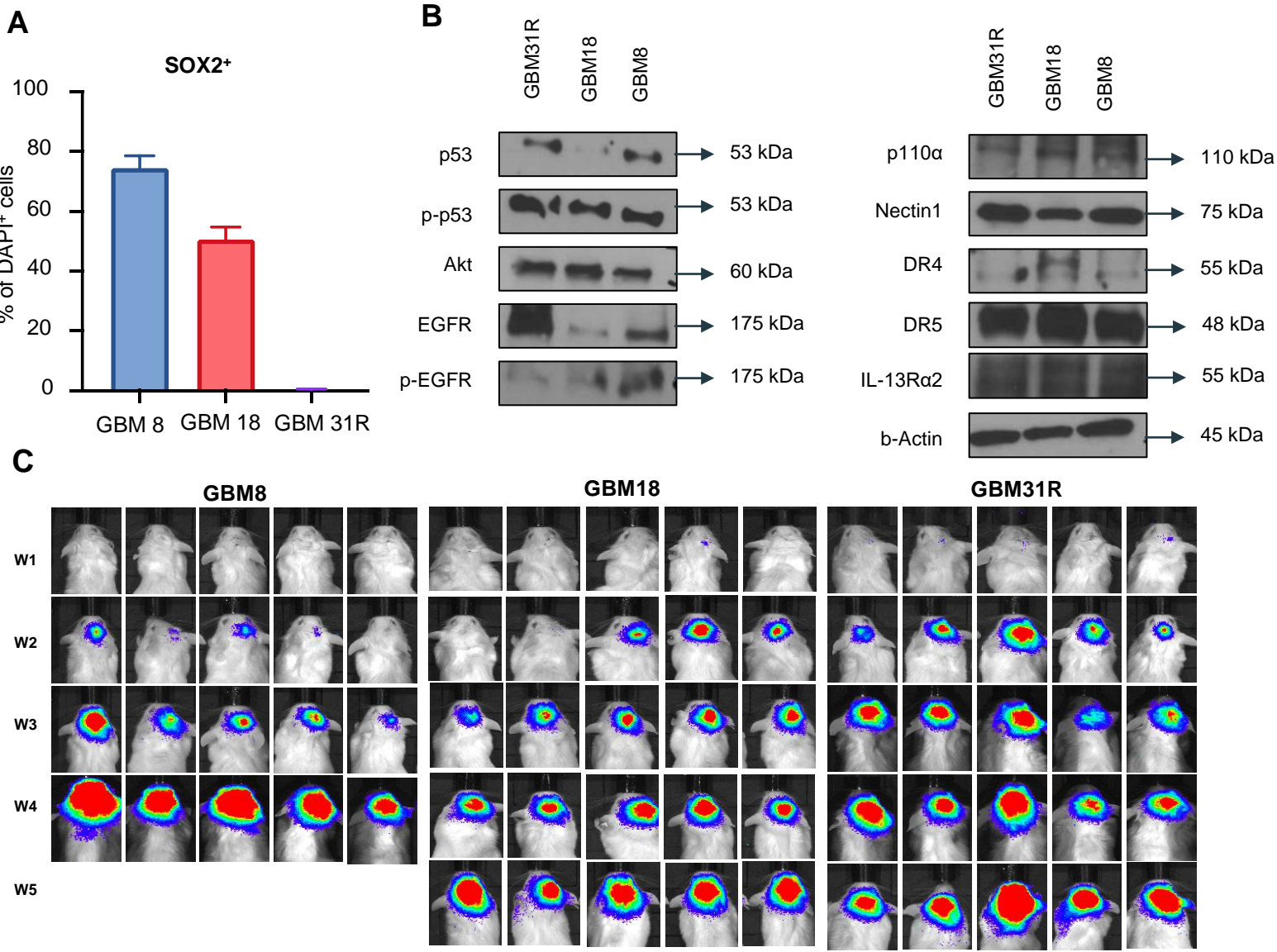

Supplement: Supplementary Figure 1 — Characterization of the patient-derived GBM cell lines. (A) Quantification of SOX2-positive cells as a ratio of DAPI-positive cells for all samples. N=6. Mean ± SEM. (B) Western blot analysis showing the expression patterns of p53, p-p53, pAKT, AKT, EGFR, pEGFR, p110a, Nectin1, DR4, DR5, and IL-13Ra2 in the cell lysates prepared from hGBM lines after in vitro culturing. B-Actin was used as a loading control. (C) Images obtained from IVIS bioluminescence imaging were used to determine tumor growth of the BLT humanized mice for three patient-derived GBM cell lines (n=5 per group). [file DataSheet_1.pdf]
